# Synthesis and Properties of Nanosized Stoichiometric Cobalt Ferrite Spinel

**DOI:** 10.3390/ma11071241

**Published:** 2018-07-19

**Authors:** Vilém Bartůněk, David Sedmidubský, Štěpán Huber, Marie Švecová, Pavel Ulbrich, Ondřej Jankovský

**Affiliations:** 1 Department of Inorganic Chemistry, Faculty of Chemical Technology, University of Chemistry and Technology, Technická 5, 166 28 Prague 6, Czech Republic; Vilem.Bartunek@vscht.cz (V.B.); David.Sedmidubsky@vscht.cz (D.S.); Stepan.Huber@vscht.cz (Š.H.); 2 Department of Analytical Chemistry, Faculty of Chemical Engineering, University of Chemistry and Technology, Technická 5, 166 28 Prague 6, Czech Republic; Marie.Svecova@vscht.cz; 3 Department of Physical Chemistry, Faculty of Chemical Engineering, University of Chemistry and Technology, Technická 5, 166 28 Prague 6, Czech Republic; 4 Department of Biochemistry and Microbiology, Faculty of Food and Biochemical Technology, University of Chemistry and Technology, Technická 5, 166 28 Prague 6, Czech Republic; Pavel.Ulbrich@vscht.cz

**Keywords:** nanoparticles, ferrites, thermal decomposition, spinels, magnetic properties

## Abstract

Nanoparticles with controllable sizes of ferrite spinel CoFe_2_O_4_ were formed by thermal treatment of cobalt-iron glycerolate. Thermal behavior during the heating was studied by differential thermal analysis combined with thermogravimetry. The precursor, as well as the prepared nanoparticles, were analyzed by a broad spectrum of analytic techniques (X-Ray photoelectron spectroscopy (XPS), X-Ray diffraction (XRD), Energy dispersive spectroscopy (EDS), Atomic absorption spectroscopy (AAS), Scanning electron microscopy (SEM), and Raman spectroscopy). The particle size of nanoparticles was obtained from Transmission electron microscopy and also calculated using Scherrer formula. A vibrating sample magnetometer (VSM) in a Physical Property Measurement System was used to analyze the magnetic properties of nanoparticles.

## 1. Introduction

As a part of the ferrite family, magnetic CoFe_2_O_4_ spinel is in the focus of research because of its potential use in various applications. Due to its magnetic properties, CoFe_2_O_4_ nanoparticles may be employed in catalysts [1,2], cathode electrocatalyst of microbial fuel cells [3], or in various functional composite materials [4,5], including advanced adsorbents, for the removal of anionic pollutants from water [6]. Cobalt ferrite has been also considered as a good candidate for biomedical applications [7,8,9], especially for hyperthermia treatment, because of its high magneto-crystalline anisotropy [7].

Nanosized ferrites may be synthetized in many ways. A usual synthetic technique for preparation of magnetic CoFe_2_O_4_ nanoparticles is the co-precipitation method [1,2,3,4,5,6,7,8,9,10,11,12,13], but also hydrothermal [14,15,16,17], sol-gel auto-combustion [18,19], and microwave assisted [20,21] methods can be applied.

Thermal treatment of glycerol-based organometallic compounds (glycerolates) can be used for synthesis of nanoparticles with variable sizes, e.g., cobalt oxides [22], iron oxide [23], manganese oxides [24,25], and chromium oxide [26]. In addition, mixed ferrites, such as NiFe_2_O_4_ [27], have been synthetized and, through the use of a reduction atmosphere, some pure metallic nanoobjects, e.g., cobalt nanofoam [28], can be prepared. As a solvent, template compound or chelation agent, e.g., glycerol, is particularly advantageous. It exhibits a high boiling point and its viscosity is notably dependent on temperature and water dilution, therefore it is easily controllable for the purpose of nanoparticle stabilization. Furthermore, it is extra affordable in comparison with similar compounds and accessible in large quantities—therefore, the methods relying on glycerol-based precursors may have a good outlook for large-scale industrial application.

In our contribution, we focused on the synthesis of CoFe_2_O_4_ nanoparticles. Cobalt-iron glycerolate and CoFe_2_O_4_ nanoparticles were analyzed in detail by a broad spectrum of analytic techniques. The main focus was given to magnetic properties of CoFe_2_O_4_ spinel.

## 2. Materials and Methods 

Cobalt-iron glycerolate was synthesized by a reaction of glycerol (in excess) with Fe(NO_3_)_3_ and Co(NO_3_)_2_ mixed in the target molar proportion 2:1. The synthesis was performed under reflux of glycerol (boiling, 290 °C) for four hours without using any additional atmosphere. After the heat treatment, the mixture was put into water and the solid phase was subsequently obtained by centrifugation. The obtained solid material was washed using distilled water and ethanol and dried. The prepared glycerolate was labeled as Co-Fe-GLY. The synthesized cobalt-iron glycerolate was thermally decomposed in the tube furnace in a dynamic air atmosphere (*p*_O2_/*p*^0^ = 0.21, *p*^0^ = 101 kPa). The temperatures applied were the following: 500 °C, 600 °C, 700 °C, or 800 °C for 1 minute (rates 10 K min^−1^). The prepared nanoparticles were termed as Co-Fe-500, Co-Fe-600, Co-Fe-700, and Co-Fe-800, accordingly.

X-Ray diffraction (XRD) patterns were measured using Bruker D2 Phaser (Bruker, Karlsruhe, Germany) powder diffractometer (Bragg-Brentano geometry) using CoKα radiation (λ = 0.1789 Å, U = 40 kV, I = 30 mA).

X-Ray photoelectron spectroscopy (XPS) was measured with ESCAProbeP (Omicron Nanotechnology Ltd, East Grinstead, UK) spectrometer using a monochromatic aluminum X-ray radiation source (1486.7 eV).

Scanning electron microscopy (SEM) with a FEG electron source (Lyra, Tescan, Brno, Czech Republic) was used to investigate the morphology. Elemental composition was performed using an Energy dispersive spectroscopy (EDS) analyzer (X-Max^N^) with a 20 mm^2^ SDD (Silicon Drift Detector) (X-Max^N^, Oxford instruments, Abingdon-on-Thames, UK) and AZtecEnergy software (v 2.1, Oxford instruments, Abingdon-on-Thames, UK). Measurements were carried out using 10 kV electron beam.

Atomic absorption spectroscopy (AAS) was used to determine the concentration of metal elements using Agilent 280FS AA device (Agilent Technologies, Mulgrave, Australia) with a flame-atomization technique. The used wavelengths were 240.7 nm for cobalt and 248.3 nm for iron. Acetylene-air flame was used for the measurement.

Raman spectroscopy was measured using confocal Raman microspectrometer Renishaw inVia Reflex (Renishaw, Wotton under Edge, UK) equipped with a diode laser (excitation line 785 nm).

The thermal behavior was probed by simultaneous thermal analysis (STA) using Linseis STA PT1600 apparatus (Linseis Messgeraete GmbH, Selb, Germany) with the heating rate 10 K min^−1^ in dynamic air atmosphere.

Transmission electron microscopy (TEM) was performed using microscope JEOL JEM-1010 (JEOL, Tokyo, Japan) at an accelerating voltage of 80 kV. The micrographs were acquired by SIS MegaView III digital camera (Soft Imaging Systems, Münster, Germany) and analyzed by means of AnalySIS v. 2.0 software.

Vibrating sample magnetometer (VSM) installed in Physical Property Measurement System (PPMS, Quantum Design, San Diego, CA, USA) was used for the measurements of the magnetization curves (μ_0_*H* = +7 to −7 T, *T* = 4.5 and 300 K) and for the magnetic susceptibility in the temperature range 4.5–340 K (applied magnetic field μ_0_*H* = 0.1 T). The field cooled (FC) susceptibility was acquired on cooling in the magnetic field in a sweeping mode (cooling rate 5 K min^−1^), while the zero-field cooled (ZFC) susceptibility was measured on heating (heating rate 5 K min^−1^) after cooling the sample in zero field and then switching on the field. The vibrating frequency was 60 Hz and the amplitude 1 mm.

## 3. Results and Discussion

**Figure 1:**
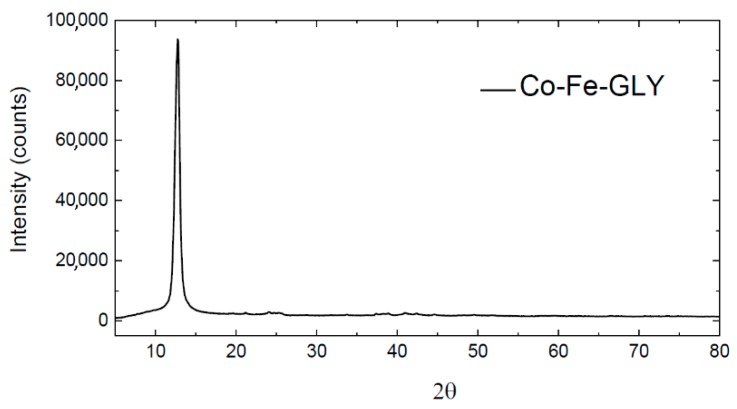
Diffractogram of Co-Fe-GLY.

The successful synthesis of cobalt-iron glycerolate (Ni-Fe-GLY) was confirmed by XRD. The observed XRD pattern is shown in Figure 1. Similar to manganese glycerolate [24], a wide reflection (at 2*θ* = 12.78) was observed, confirming that the synthetized glycerolate was highly non-crystalline or nanostructured.

**Figure 2:**
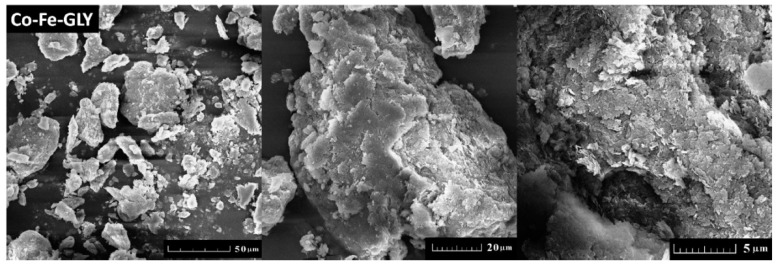
SEM micrographs of Co-Fe-GLY obtained at various magnifications.

**Figure 3:**
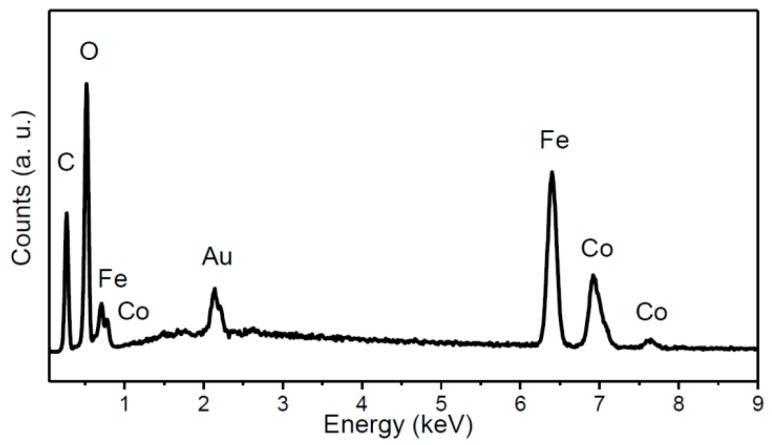
EDS spectrum of Co-Fe-GLY.

Cobalt-iron glycerolate was studied using SEM and EDS. Large agglomerates with a size over 20 µm were found; only few particles were of sub-micron size (see Figure 2). Let us note that a similar structure has been also obtained for other glycerolates [22,23,24]. SEM-EDS spectrum is shown in Figure 3, confirming the presence of carbon, oxygen, iron, and cobalt. Gold, originating from the sputtering, was also detected. The obtained composition Co_1_Fe_2.02_C_10.7_O_7.76_ (Co stoichiometry was fixed to 1) was determined as an average from four measurements. The corresponding C/O ratio was ~1.38:1, which was further confirmed by AAS. Similarly, the ratio of metals in cobalt-iron glycerolate 1:2.08 as obtained from AAS is in line with SEM-EDS results.

**Figure 4:**
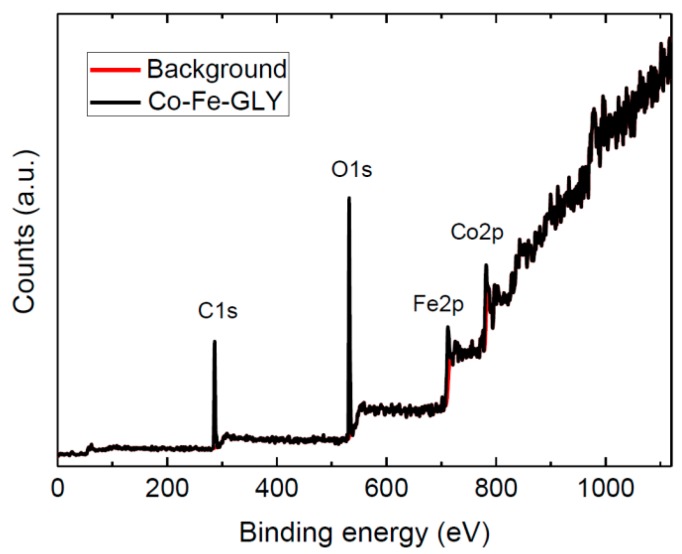
XPS survey spectrum of Co-Fe-GLY.

The Co-Fe-GLY composition was examined by XPS (Figure 4). The Co2p, Fe2p, C1s, and O1s peaks were identified in the obtained survey spectrum. The first peak corresponding to C1s was obtained at ~286.5 eV. A peak corresponding to O1s was found at ~531.6 eV. A third peak observed at ~712.5 eV can be attributed to Fe2p. The last peak was found at ~782.4 eV, this Co2p peak confirmed the presence of cobalt in the glycerolate. The observed positions for Co2p and Fe2p are in good agreement with the literature [29]. Chemical composition Co_1_Fe_1.80_C_42.06_O_25.57_ (Co being fixed to 1) was calculated from the spectrum. The C/O ratio ~1.64:1 was slightly higher to that determined by SEM-EDS. The composition of bulk cobalt-iron glycerolate probed by XPS is slightly different compared to EDS results due to a surface sensitivity of XPS.

**Figure 5:**
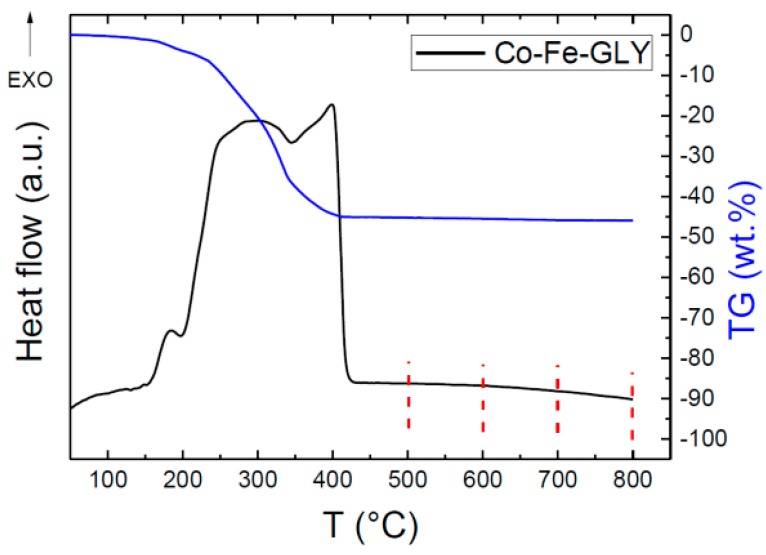
STA analysis of Co-Fe-GLY with marked decomposition temperatures.

Thermal behavior was studied by thermal analysis. As seen from Figure 5, one exothermic effect was observed; however, this effect is clearly composed of three partial transitions. The first exo-peak started at ~150 °C and reached its maximum at 185 °C, the second major exo-effect reached the maximum at 298 °C, while the third one was indicated at 399 °C. The thermal decomposition led to a formation of pure nanoparticles, which will be discussed later. The decomposition/oxidation was accompanied be a weight loss of ~45 wt. %. Assuming the formation of pure CoFe_2_O_4_, we can estimate the glycerolate molar mass as 425.6 g mol^−1^, corresponding well with the chemical composition determined by EDS.

**Figure 6:**
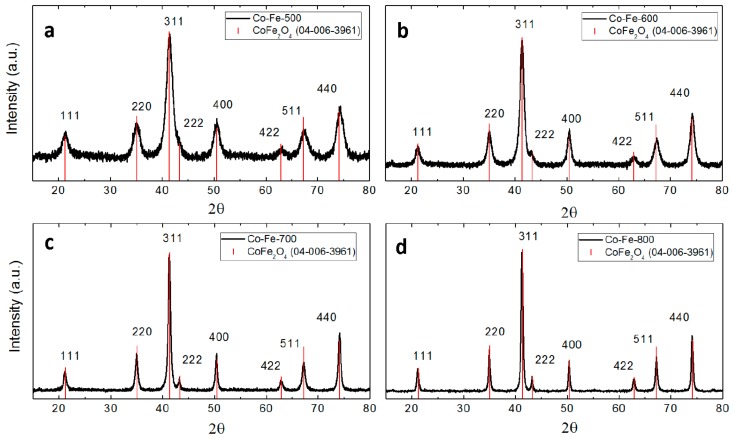
XRD patterns of prepared nanoparticles: (**a**) Co-Fe-500, (**b**) Co-Fe-600, (**c**) Co-Fe-700 and (**d**) Co-Fe-800.

Based on the XRD analysis, it can be confirmed that nanocrystalline CoFe_2_O_4_ was obtained (Figure 6) [30]. Next, Scherrer formula was used to determine the crystallite sizes. The results confirmed the expected behavior: At higher temperatures, nanocrystal growth took place, hence bigger nanoparticles were formed. The calculated crystallite sizes of CoFe_2_O_4_ were ~6.2 nm, ~9.7 nm, ~17.5 nm, and ~28.2 nm for the samples Co-Fe-500, Co-Fe-600, Co-Fe-700, and Co-Fe-800, respectively. The evolution of particle size with temperature is obvious. The acquired diffraction patterns are in good agreement to the literature [29,31].

**Figure 7:**
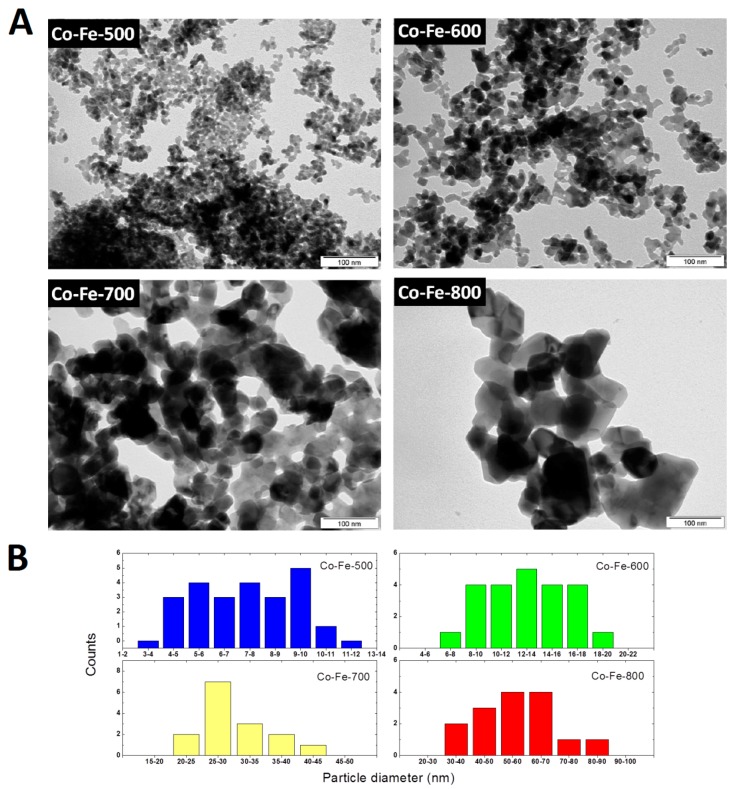
(**A**) TEM micrographs of synthetized nanoparticles; (**B**) particle size distribution obtained by image analysis.

Transmission electron microscopy (TEM) was applied in order to prove the calculated particle diameters (Figure 7). While the nanoparticles Co-Fe-500 and Co-Fe-600 are ultrafine with homogenous particle size distribution, the other two samples prepared at higher temperatures significantly differed due to crystallite growth resulting in larger nanoparticles with different shapes and divergent particle sizes. However, it was proved that particle diameters are growing with increasing temperature. The histograms showing the particle size distribution of the selected area are also shown in Figure 7.

**Figure 8:**
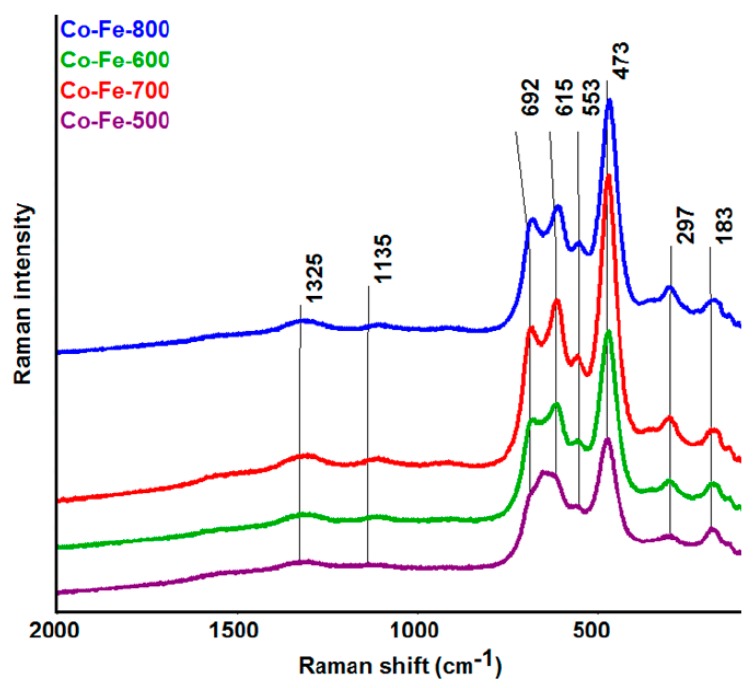
Raman spectra of CoFe_2_O_4_ nanoparticles prepared at various temperatures.

The Raman spectroscopy confirmed the presence of an inverse spinel structure Fe[CoFe]O_4_ [32,33,34], without any indication of signals belonging to impurity phases, such as those corresponding to precursors. The Raman spectra are shown in Figure 8. The bands at 1325 and 1135 cm^−1^ are assigned to the A_1g_ and T_2g_ modes of the 2nd order. The next bands at 692 cm^−1^ and at 615 cm^−1^ correspond to A_1g_ symmetric stretching (tetrahedral breath mode) of oxygen atoms with respect to Fe and Co ions. T_2g_ modes (asymmetric stretching and bending, respectively) are assigned to bands at the 553 and 473 cm^−1^, and the T_2g_ mode at 183 cm^−1^ belongs to a translation motion of the whole tetrahedron. The band at 297 cm^−1^ is assigned to E_g_ symmetric bending of Fe(Co)-O. The results of Raman analysis are also summarized in Table 1.

**Figure 9:**
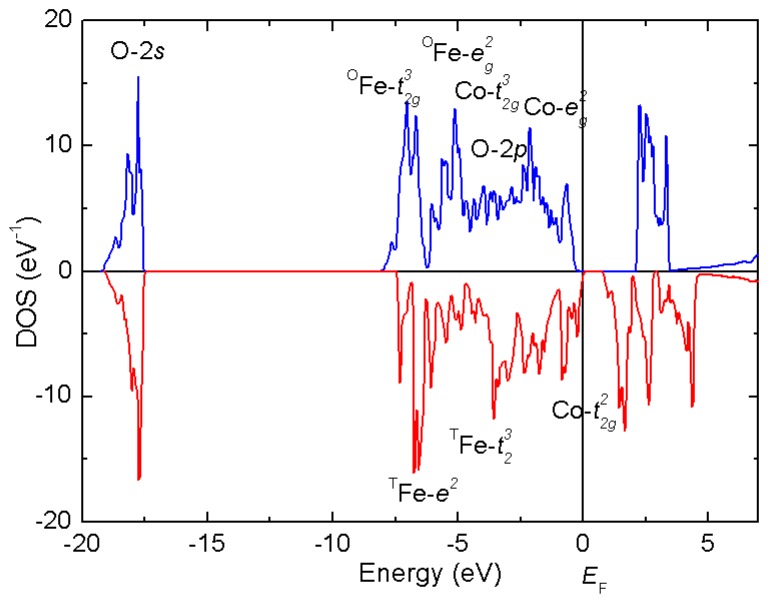
Density of states (DOS) around the Femi level *E*_F_ of the inverse spinel structure, Fe^3+^[Fe^3+^Co^2+^] O_4_. The majority spin (blue, positive): Co, Fe(oct)-*t*_2g_^3^e_g_^2^. Minority spin (red, negative): Fe(tet)-*e*^2^*t*_2_^3^, Co(oct)-*t*_2g_^2^.

CoFe_2_O_4_ in a bulk form is known to exist predominantly in the inverse spinel structure (with only 10–20 per cent of Co occupying the tetrahedral sites). Considering of a 90° superexchange interaction between both types of cations occupying the octahedral sites (Co^2+^-3*d*^7^ and Fe^3+^-3*d*^5,^ both occurring in high spin state) leading to parallel spin ordering, and an antiferromagnetic coupling between the tetrahedral (spin S = 5/2) and octahedral positions (S = 4), we can anticipate a ferrimagnetic behavior with a saturated magnetic moment *M*_sat_ ~3 μ_B_ (S = 3/2) per formula unit (f.u.). Such behavior is indeed observed below the Curie temperature *T*_C_ ~793 K. These experimental findings are in line with our DFT calculations (GGA+U, APW+lo, as implemented in Wien2k), confirming the inverse spinel structure (favored by 50 kJ mol^−1^ with respect to normal spinel) and the AF interaction between the tetrahedral Fe^3+^ and mixed Co^2+^/Fe^3+^ octahedral sites. The integrated density of states for spin-up and spin-down channel, as shown in Figure 9, yields the net magnetic moment 3 μ_B_.

**Figure 10:**
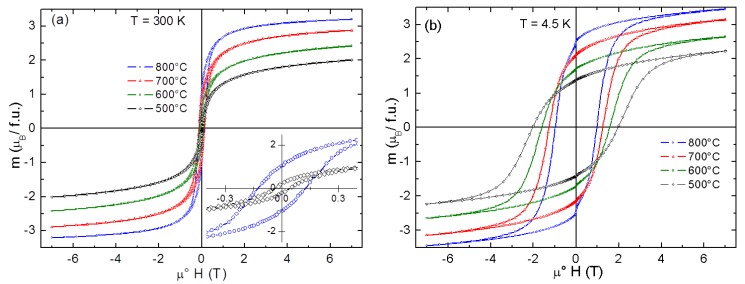
Magnetization curves of CoFe_2_O_4_ recorded at 300 K (**a**) and 4.5 K (**b**). A blow-up of two hysteresis curves (Co-Fe-500 and Co-Fe-800) is shown in the inset of panel (**a**).

The magnetization curves measured at T = 300 K and at T = 4.5 K (Figure 10) reveal a saturation, though not complete, at higher magnetic fields, which is substantially suppressed compared to the bulk form and decreases with reducing crystallite size. The main parameters of the obtained magnetization curves are summarized in Table 2.

**Figure 11:**
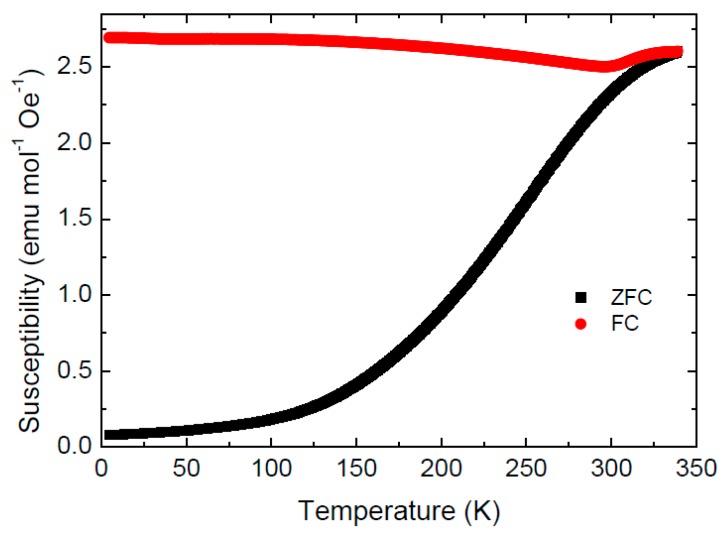
Field cooled (FC) and zero field cooled (ZFC) magnetic susceptibility of Co-Fe-500 sample measured in the temperature sweeping mode at a heating and cooling rate 5 K min^−1^.

Only a small hysteresis is observed on the magnetization curves recorded at *T* = 300 K (coercivity field ranging from μ°*H*_c_ = 45 mT to μ°*H*_c_ = 130 mT for Co-Fe-500 and Co-Fe-800, respectively). This behavior, together with a notable difference between the zero-field cooled (ZFC) and field cooled (FC) magnetic susceptibility below room temperature (see Figure 11), suggests a superparamagnetic (SPM) behavior. Superparamagnetic behavior of nanoparticles (5–8 nm) at room temperature was already identified by Repko et al. [35].

Interestingly, the FC susceptibility of Co-Fe-500 sample undergoes an upturn from the slightly decreasing trend at 300 K before merging the ZFC curve at the blocking temperature *T*_B_ ~340 K. (determined as a furcation point of the ZFC-FC curves). Blocking temperatures in the range 200–400 K have been reported for various CoFe_2_O_4_ nanoparticles [35].

At lower temperatures, the slow relaxation brings about a pronounced hysteresis, which increases with reducing particle size and reaches the highest coercivity field μ°*H*_c_ = 2 T for Co-Fe-500 and the highest remanence *M*_r_ ~2.5 μ_B_/f.u. for Co-Fe-800. Let us note that much smaller hysteresis (μ°*H*_c_ = ~120 mT) and also a lower blocking temperatures *T*_B_ have been recently found in NiFe_2_O_4_ inverse spinels [27] with comparable particle sizes. This can be in part interpreted in terms of larger magnetic anisotropy of CoFe_2_O_4_ (K_1_ = 2 × 10^6^ as compared with K_1_ = −5 × 10^4^ for NiFe_2_O_4_ [36]), bringing about a higher blocking temperature, however, magnetic frustration due to competing exchange interactions, as well as site and displacive disorder associated with the surface states, might be at the origin of this huge hysteresis.

The observed magnetic behavior makes cobalt ferrite nanoparticles excellent candidates for hyperthermia treatment and other medicinal applications. The particle size control, as demonstrated on the proposed thermal decomposition of glycerolates, is indeed essential for tailoring the nanomaterial magnetic properties

One of the main advantages of the described synthesis method of CoFe_2_O_4_ nanoparticles in comparison with co-precipitation or hydrothermal methods is the absence of other elements and ions, e.g., Na^+^ ions in the case of co-precipitation methods or various capping agents in the case of usual hydrothermal techniques. The ability to control the nanoparticles size also represents a great advantage of the present method. Moreover, the method operates with affordable, non-toxic and available precursors, and is usable even for preparation of larger amounts of the material, therefore with the possibility for further development for applications on an industrial scale.

## 4. Conclusions

Cobalt ferrite spinel nanoparticles with stoichiometry CoFe_2_O_4_ were synthesized by thermal decomposition of cobalt-iron glycerolate. Nanoparticles’ sizes were controlled by temperature of decomposition of previously synthesized glycerolate precursor in the range from 6.2 to 28.2 nm. Both precursor and product were characterized by various means, including Raman spectroscopy, XRD, SEM-EDS, TEM, AAS, and XPS. The CoFe_2_O_4_ spinel nanoparticles exhibit a pronounced hysteresis, a suppressed saturated magnetization, and high blocking temperatures due to large magnetic anisotropy and nanosizing effects. Our method may be applied for preparation of high volumes of magnetic CoFe_2_O_4_ nanoobjects of selected sizes with a surface free of surfactant or other contamination.

## Figures and Tables

**Table 1:** Measured Raman shifts of CoFe_2_O_4_ nanoparticles.

Raman Shift (cm^−1^)			Vibrational Mode	Assignment
Experimental	Wang et al. [32]	Chandramohan et al. [33]		
1325	-	-	A_1g_	2nd order bands
1135	-	-	T_2g_(2)	
692	683	695	A_1g_ (1)	symmetric stretching Fe-O
615	617	624	A_1g_ (2)	symmetric stretching Fe(Co)-O
553	563	575	T_2g_(2)	asymmetric stretching Fe-O
473	471	470	T_2g_(3)	asymmetric bending Fe(Co)-O
297	300	312	E_g_	symmetric bending Fe(Co)-O
183	188	210	T_2g_(1)	translation motion of the whole tetrahedron

**Table 2:** Coercivity filed μ°*H*_c_, remanent magnetization *M*_r_, magnetic moment at the maximum field M_7T_ obtained from the magnetization curves measured at 300 K and 4.5 K, and the blocking temperature *T_B_* determined as a furcation point between field cooled and zero-field cooled (FC and ZFC) curves of magnetic susceptibility.

Co-Fe	μ°*H*_c_ (300 K) mT	*M*_r_ (300 K) μ_B_/f.u.	M_7T_ (300 K) μ_B_/f.u.	μ°*H*_c_ (4.5 K) T	*M*_r_ (4.5 K) μ_B_/f.u.	M_7T_ (4.5 K) μ_B_/f.u.	*T_B_* K
500	45	0.20	2.00	1.95	1.36	2.23	340
600	90	0.51	2.41	1.58	1.72	2.65	328
700	138	0.81	2.89	1.28	2.13	3.15	300
800	130	1.20	3.20	0.99	2.47	3.46	300
